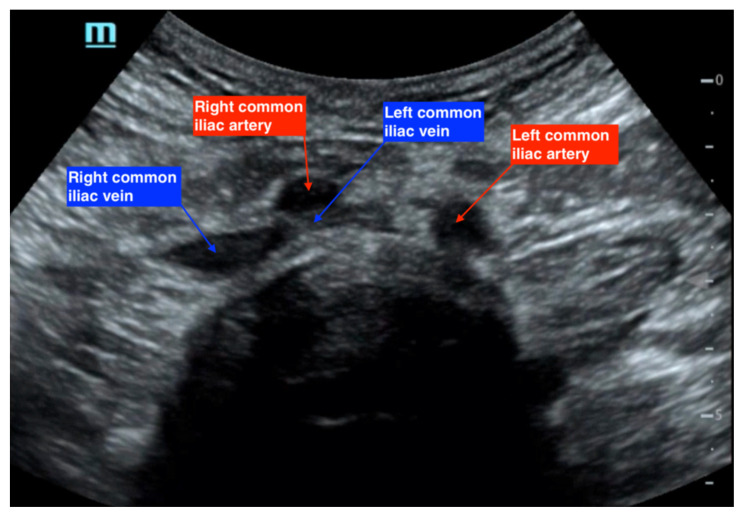# A Case Report of May-Thurner Syndrome Identified on Abdominal Ultrasound

**DOI:** 10.21980/J8C64K

**Published:** 2022-07-15

**Authors:** Michelle Brown, Edmund Hsu, Christopher McCoy, Matthew Whited

**Affiliations:** * University of California, Irvine, Department of Emergency Medicine, Orange, CA

## Abstract

May-Thurner syndrome (MTS) is most commonly caused by the compression of the left iliac vein by the right iliac artery against the lumbar spine, which leads to the development of a partial or occlusive deep venous thrombosis (DVT).^1^ Diagnosis begins with a duplex ultrasound of the lower extremities to evaluate for a femoropopliteal thrombus, and in high-risk patients where a more proximal DVT is suspected and the DVT ultrasound is negative, a computed tomography venogram (CTV) or magnetic resonance venogram (MRV) is performed.^1^,^3^ In this case report, a patient presented to the emergency department (ED) with two days of left lower extremity pain and swelling. Initial lower extremity DVT ultrasound was negative, so a CTV was ordered and revealed a thrombus in the left common iliac vein with overlying compression by the right common iliac artery, suggesting the diagnosis of May-Thurner syndrome (Figure 1). Afterwards, a point-of-care ultrasound (POCUS) was performed at bedside to evaluate the caval and iliac arteries and the findings were consistent with the CTV (Figure 2, 3, 4). If the POCUS was performed prior to the CTV, the patient may have been spared the radiation exposure from CT, as well as the risks of intravenous (IV) contrast required for a venogram. Therefore, in high risk patients in whom a negative DVT ultrasound will prompt advanced imaging with CTV or MRV, I propose the addition of a lower abdominal ultrasound using a curvilinear probe to assess the caval and iliac arteries prior to obtaining a CTV or MRV.

**Topics:**

May-Thurner Syndrome, leg swelling, POCUS, ultrasound, deep venous thrombosis.

## Brief introduction

May-Thurner syndrome (MTS) most commonly results from an anatomic variation in which the right iliac artery compresses the left iliac vein, resulting in a partial versus occlusive deep venous thrombosis.^1^ Risk factors include younger age, typically between the ages of twenty to forty years old, female, hormone use, pregnancy, recent surgery or immobilization, and prior iliac stenting.^1^,^2^ Though many patients with anatomic variability of MTS are asymptomatic, those who present to the hospital after developing symptoms classically present with acute onset pain and swelling of the left leg, venous claudication, or chronic symptoms of venous insufficiency.^1^ The patient is often a woman in her twenties or thirties with the chief complaint of swelling to her left lower extremity for four to six weeks.^1^,^2^ Physical exam may reveal swelling that involves the entire limb.^1^ Patients who present with unilateral leg swelling will often undergo a DVT ultrasound, followed by a computed tomography venogram (CTV) or magnetic resonance venogram (MRV) to assess the iliac vessels.^1^,^3^ Although MTS is not a common diagnosis made in the emergency department (ED) and is more frequently diagnosed as an outpatient, a high index of suspicion for an underlying proximal thrombosis may prompt the ED physician to obtain further advanced imaging upon receiving a negative DVT ultrasound. In this case report, a patient was found to have evidence of May-Thurner syndrome on a bedside point-of-care abdominal ultrasound (POCUS), and a CTV revealed the extent of the thrombosis. Although the CTV was performed prior to the ultrasound, there is evidence of MTS on the abdominal POCUS. As a result, it may be possible to employ ultrasound instead of CT for patients who may not be able to tolerate the contrast load due to chronic kidney disease or anaphylaxis to contrast media, or those who may not want to risk radiation exposure, such as those who are pregnant.^4^,^5^

## Presenting concerns and clinical findings

A 49-year-old Hispanic female presented to the emergency department with two days of acute pain and swelling to her left leg that she first noticed while she was walking. She reported the pain and swelling extended from her thigh down to her toes, and described it as a constant tightening sensation and rated it as a seven out of ten in severity. Her symptoms were associated with numbness and tingling throughout her leg as well as pain with ambulation. She denied any trauma, weakness, or skin changes to the leg. The only clinician she had seen for this complaint thus far was from a clinic at an outside hospital earlier that day, who referred her to an emergency department for evaluation.

On further assessment, the patient revealed that she had no medical diagnoses, no surgical history, no allergies to medication, and no current medication use or oral contraceptive use. She denied any family history of clotting disorders and any tobacco, alcohol, or drug use. Her initial vital signs included a blood pressure of 146/74, a pulse of 102, a temperature of 98.4˚F, a respiratory rate of 18, and blood oxygen saturation of 100% on room air. The physical exam was remarkable for +1 pitting edema of the left leg that extended up her left thigh, with obvious enlargement of her left lower extremity compared to her right lower extremity. She also had decreased range of motion of the entire left lower extremity due to pain. Her neurovascular exam revealed subjective decreased sensation throughout all dermatomes of the left leg, full muscle strength to her bilateral lower extremities, and equal and strong dorsalis pedis pulses bilaterally. Her skin showed no evidence of erythema, fluctuance, or crepitus. Based on her history and physical exam, appropriate laboratory testing and imaging were ordered and are further discussed below.

Given the history and physical of this patient, the differential diagnosis included venous insufficiency, deep venous thrombus, and iliac vein compression due to anatomic variability (aka May-Thurner syndrome) versus compression due to a pelvic mass, uterine enlargement, or aortoiliac aneurysm.^1^,^6^ Radiographic modalities used to diagnose May-Thurner syndrome include CT venography or MR venography.^1^ Catheter-based venography (the gold standard for diagnosis) and intravascular ultrasound (IVUS) are two more invasive alternatives when suspicion remains high in the presence of diagnostic uncertainty or anticipated treatment and can be done on an outpatient basis or after the patient is admitted to the hospital for further work-up. 1,3

## Significant findings

**Figure 1:**
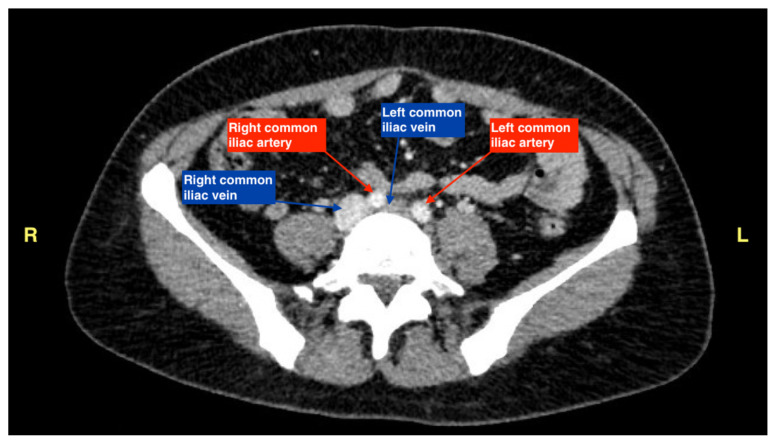


The patient initially received a venous doppler ultrasound that showed no evidence of a right or left femoropopliteal venous thrombus. Due to the high suspicion of a DVT given the symmetric swelling to the entire limb and acute onset of pain, a CTV was ordered. The transverse view of the CTV showed chronic thrombotic occlusion of the proximal left common iliac vein associated with compression from the right common iliac artery (figure 1, transverse image of CTA), as well as thrombotic occlusion of the left internal iliac vein tributary and corresponding left ascending lumbar vein. Given the previously mentioned clinical context, these features suggested the diagnosis of May-Thurner syndrome.

**Figure 2:**
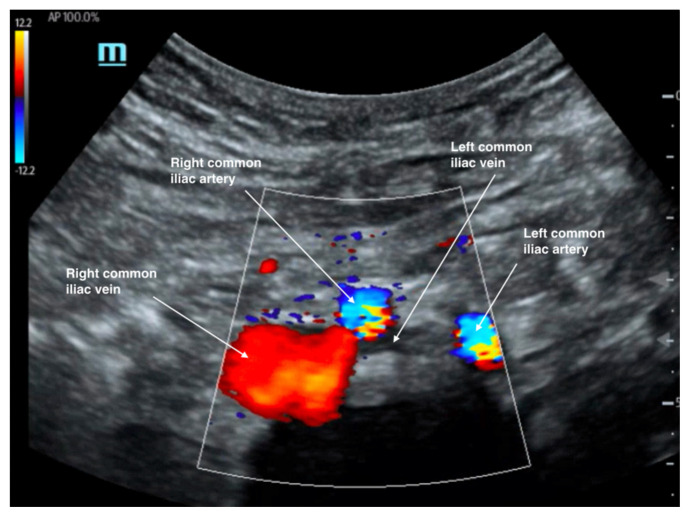


**Figure 3:**
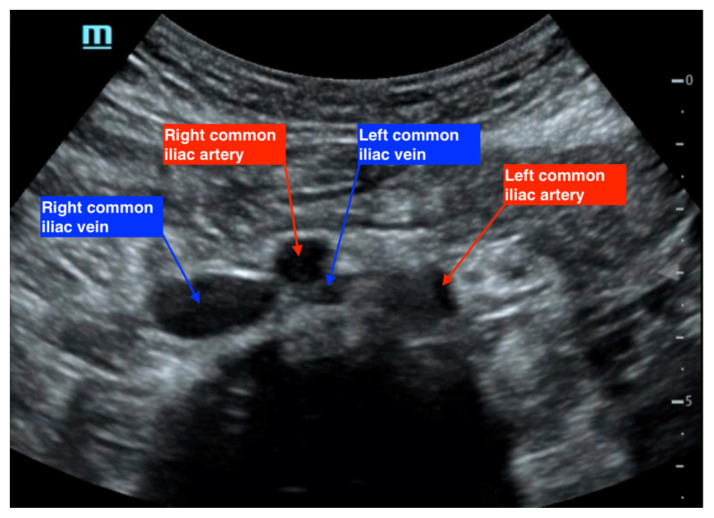


As a result of these findings on CTV, a bedside point-of-care ultrasound was performed using a curvilinear probe to assess the caval and iliac vessels, as shown in figures 2, 3, and 4. A curvilinear probe was placed on the periumbilical region of the patient in a transverse plane to evaluate the inferior vena cava (IVC) and follow the IVC down to the bifurcation of the common iliac veins. The aorta was also identified and traced to the bifurcation of the common iliac arteries. Color flow doppler was used to confirm the flow in the arterial and venous system, and demonstrates the absence of flow across the left common iliac vein (figure 2). Figure 3 highlights the anatomy of the iliac vessels with subsequent anterior to posterior compression along the transverse axis shown in figure 4, which demonstrates the lack of compressibility within the left common iliac vein, indicating the presence of a thrombus. Additionally, it is noted on these ultrasound images that the right common iliac artery can be seen directly overlying the left common iliac vein, which has a narrow diameter and suggests the diagnosis of May-Thurner syndrome (figures 2, 3, and 4). Arterial and venous doppler waveforms were not performed, but should be included in future ultrasound examinations to further support the diagnosis.

## Discussion

Duplex ultrasound of the abdomen is under-utilized in the ED and has the potential to diagnose MTS. By discussing the benefits and limitations of CTV and MRV, followed by a review of the POCUS images obtained from this patient, it becomes clear that POCUS can diagnose MTS and should be more frequently performed in the ED.

Duplex ultrasound may allow for the identification of a venous thrombosis in the iliac and femoropopliteal vessels using compression and observing the lack of compressibility of the vein.^5^ When assessing the femoral vein, a lack of respiratory variations and the absence of a response to a Valsalva maneuver can signify a more proximal obstruction.^3^,^6^ Reported sensitivity and specificity for detecting a DVT is approximately 91 and 99 percent.^1^ When assessing the iliac vessels, however, limitations include large body habitus, overlying bowel gas in the abdomen, and operator proficiency.^2^,^4^ The benefits of an ultrasound include a low cost, wide availability in emergency departments, and short duration of the exam. Ultrasound also does not expose patients to IV contrast or radiation exposure associated with CT imaging. Of note, when discussing ultrasound to assess for the presence of a venous thrombosis, the discussion rarely includes the evaluation of the caval or iliac vessels.

Unlike ultrasound, CTV and MRV identifies the presence of a thrombus by using contrast enhancement to reveal filling defects.^5^ CTV and MRV may provide the degree, severity, and chronicity of the thrombus, as well as identify collateral vessels, anatomic variations, and other extrinsic factors that could be causing compression.^1^ Sensitivity and specificity for both CTV and MRV for diagnosing MTS are greater than 95 percent.^1^ Limitations to CTV, however, include cost, allergies to intravenous contrast, high radiation exposure, and the risk of developing contrast-induced nephropathy for patients who have a history of chronic renal disease.^5^ Like CTV, MRV also allows for the detection of a venous thrombus. However, limitations include high cost, contrast-related complications, and patient anxiety due to the long duration of the exam in a confined space.^4^,^5^ MRV is rarely used for the diagnosis of a DVT due to the high expense, lack of availability, and extended duration of the exams compared to other imaging modalities.^4^

Based on the information provided by the images on POCUS as shown in the images in the appropriate clinical context, our patient would likely have been able to forgo the CTV and be directly admitted to medicine, initiated on a heparin drip, and prepared for vascular intervention. The CTV did not provide any additional information that would not have been discovered under IVUS by vascular surgery. As a result, if the POCUS of the patient’s abdomen would have been done initially alongside the DVT ultrasound of the lower extremities, the CTV may have been avoided, preventing the patient from an expensive exam, unnecessary contrast and radiation exposure, and decreasing the patient’s time to admission.

For patients with a high suspicion for a deep venous thrombosis, I propose a change to the standard diagnostic tests obtained in the emergency department. For those in whom a negative DVT ultrasound will prompt a CTV or MRV, I suggest an additional component be added to the standardized lower extremity DVT ultrasound. In these cases, if the ultrasound technician does not readily notice a compression defect in the lower extremities during the exam, if specified to do so by the physician in high-risk cases, the ultrasound technician should complete their lower extremity DVT ultrasound, followed by the evaluation the caval and iliac vessels as well. If no additional compression abnormality or abnormal doppler waveform are noted on ultrasound evaluation due to body habitus, overlying gas, or an incomplete exam, then a CTV or MRV may be pursued.^4^ However, if the sonographer is fortunate enough to identify an obvious compression abnormality or abnormal doppler waveform, the physician may decide to forego the CTV or MRV and initiate treatment.

In conclusion, ultrasound examination using the curvilinear probe to evaluate the caval and iliac vessels is under-utilized in the emergency department in the diagnosis of May-Thurner syndrome. By implementing bedside POCUS by the emergency physician or by instructing the ultrasound technician to evaluate the caval and iliac vessels on a patient in whom the DVT ultrasound is negative and suspicion remains high for a proximal DVT, the diagnosis may be found sooner, and high-cost imaging modalities that expose patients to IV contrast and radiation may be avoided entirely and allow the patient to proceed directly to treatment and intervention.

## Supplementary Information

















## Figures and Tables

**Figure 4:**