# Supplementary figures and images for: A Case Report of May-Thurner Syndrome Identified on Abdominal Ultrasound
Source: J Educ Teach Emerg Med. 2022 Jul 15;7(3):V14–9. doi: 10.21980/J8C64K (PMC10332705; doi:10.21980/J8C64K)

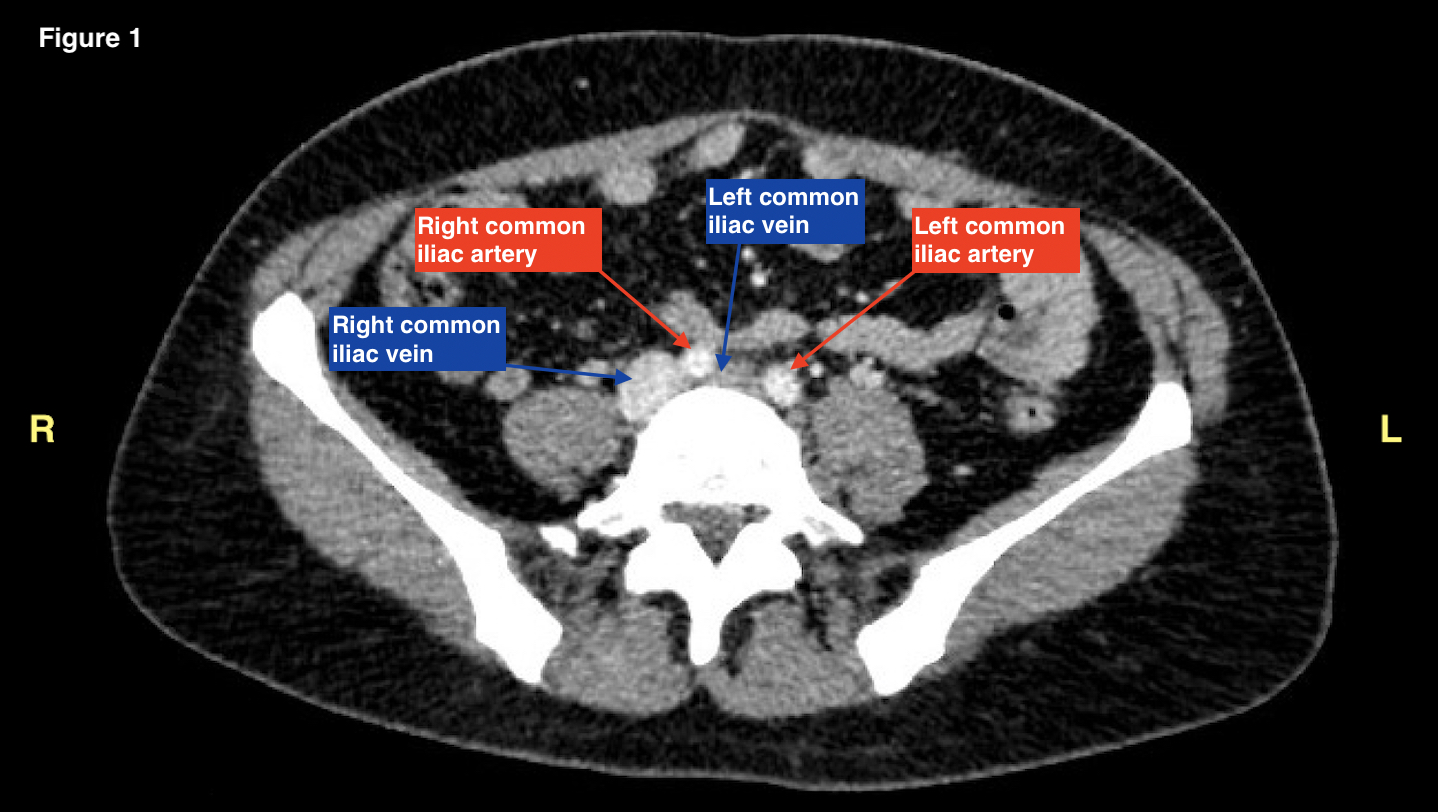

Supplement: Supplementary file 1 [file jetem-7-3-v14-supp1.jpeg]

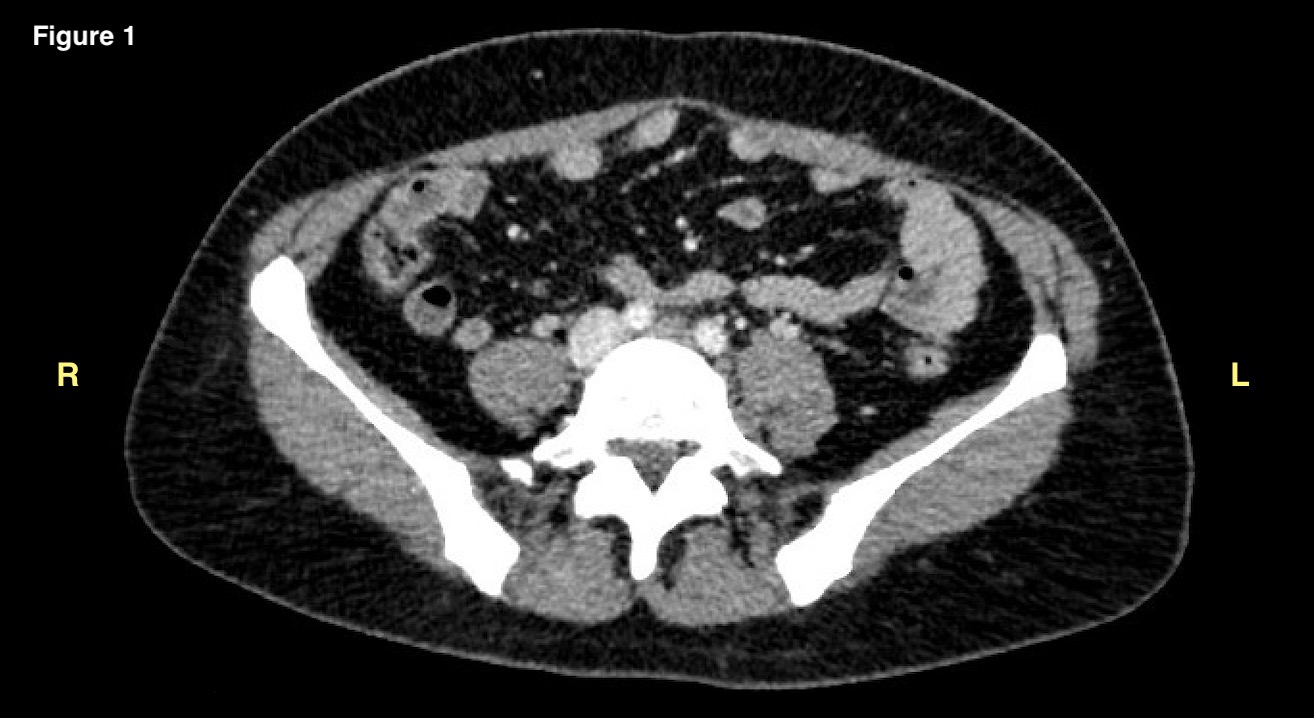

Supplement: Supplementary file 2 [file jetem-7-3-v14-supp2.jpeg]

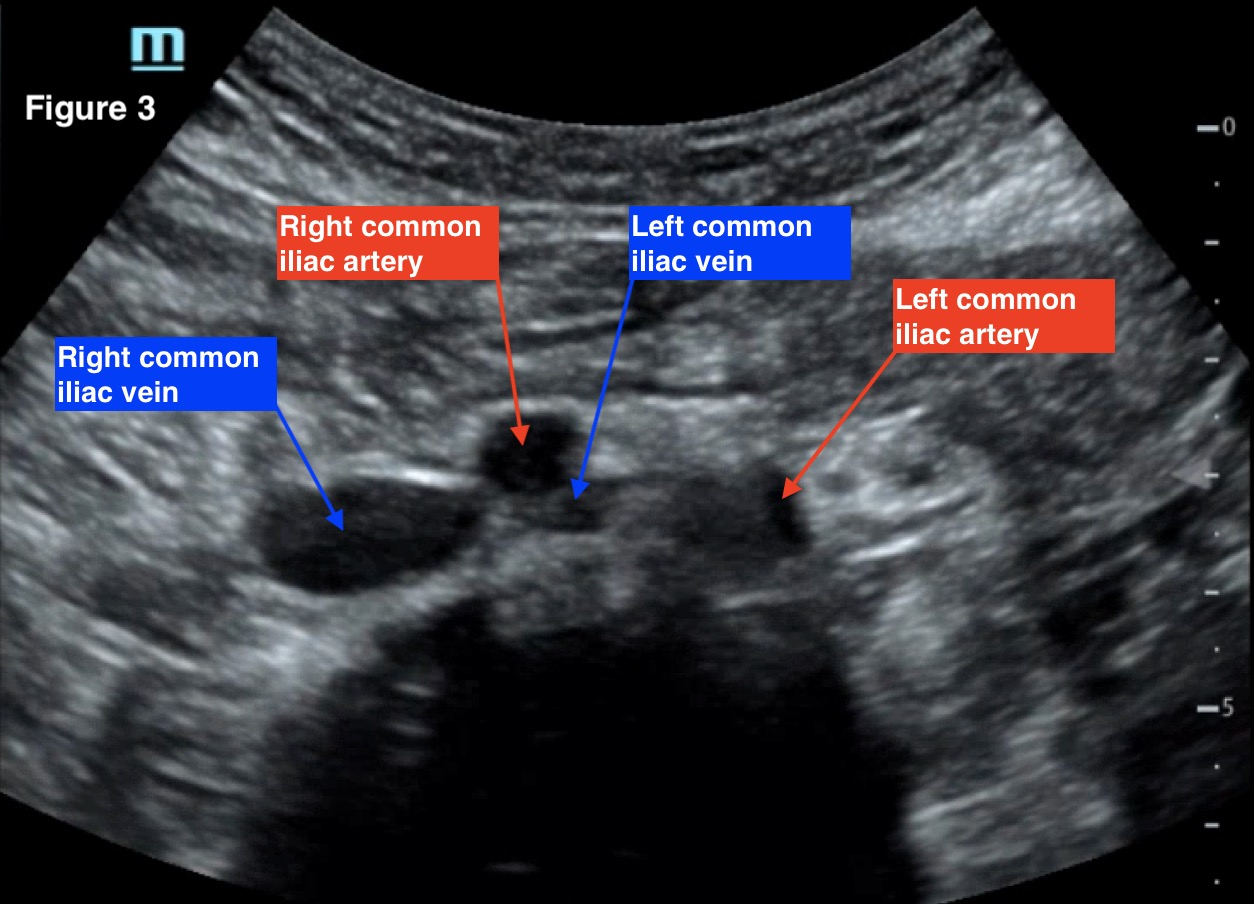

Supplement: Supplementary file 3 [file jetem-7-3-v14-supp3.jpeg]

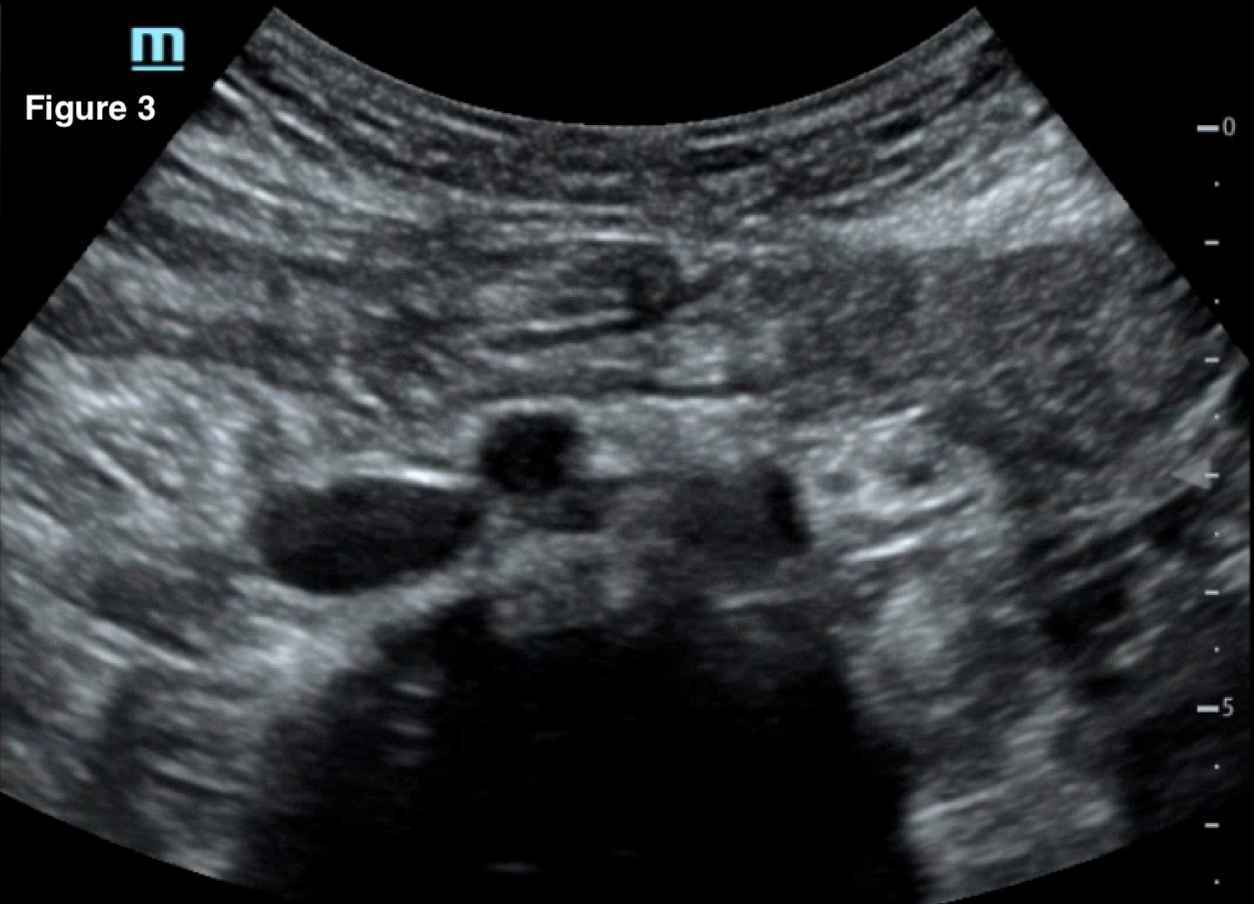

Supplement: Supplementary file 4 [file jetem-7-3-v14-supp4.jpeg]

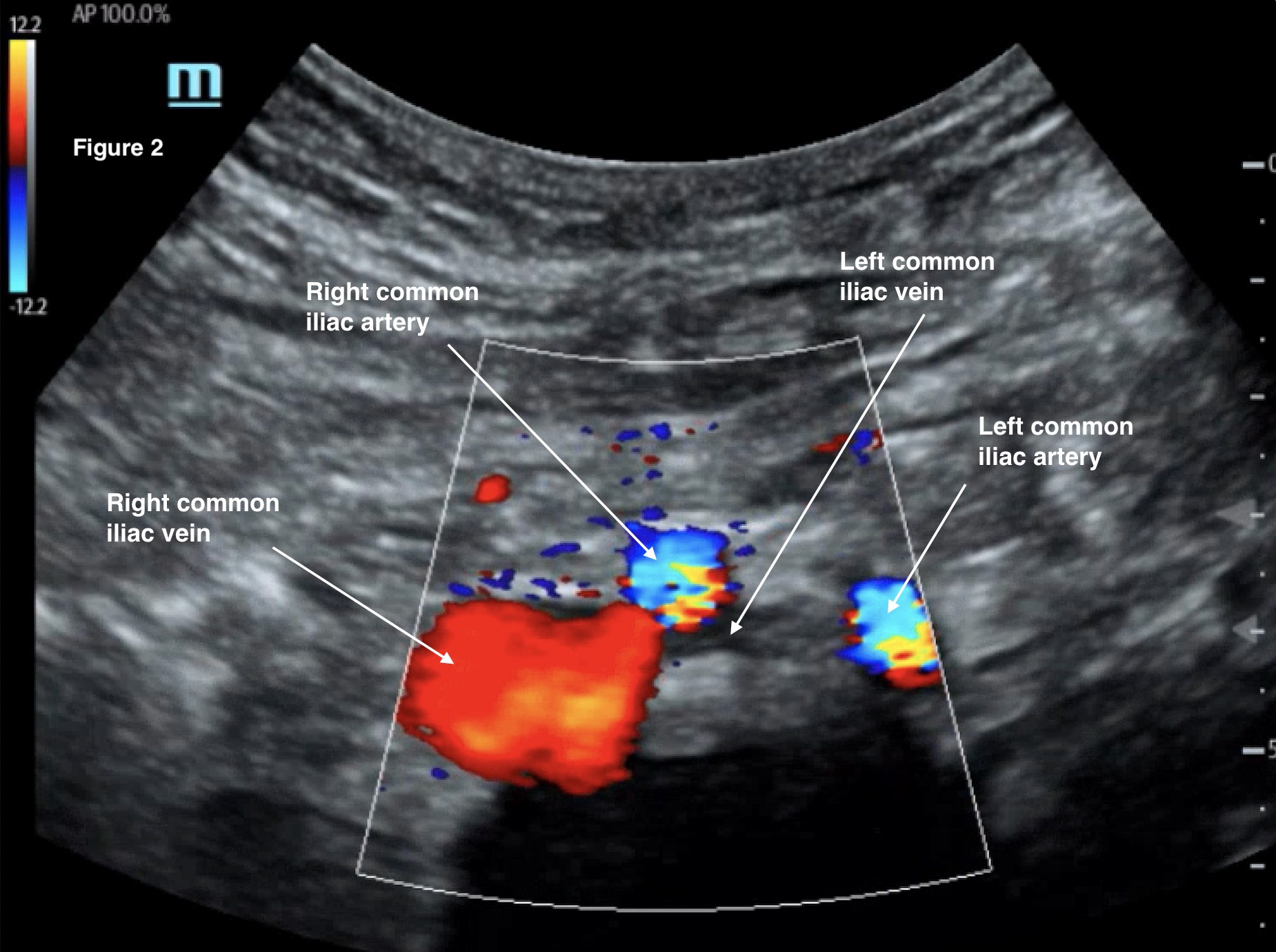

Supplement: Supplementary file 5 [file jetem-7-3-v14-supp5.jpeg]

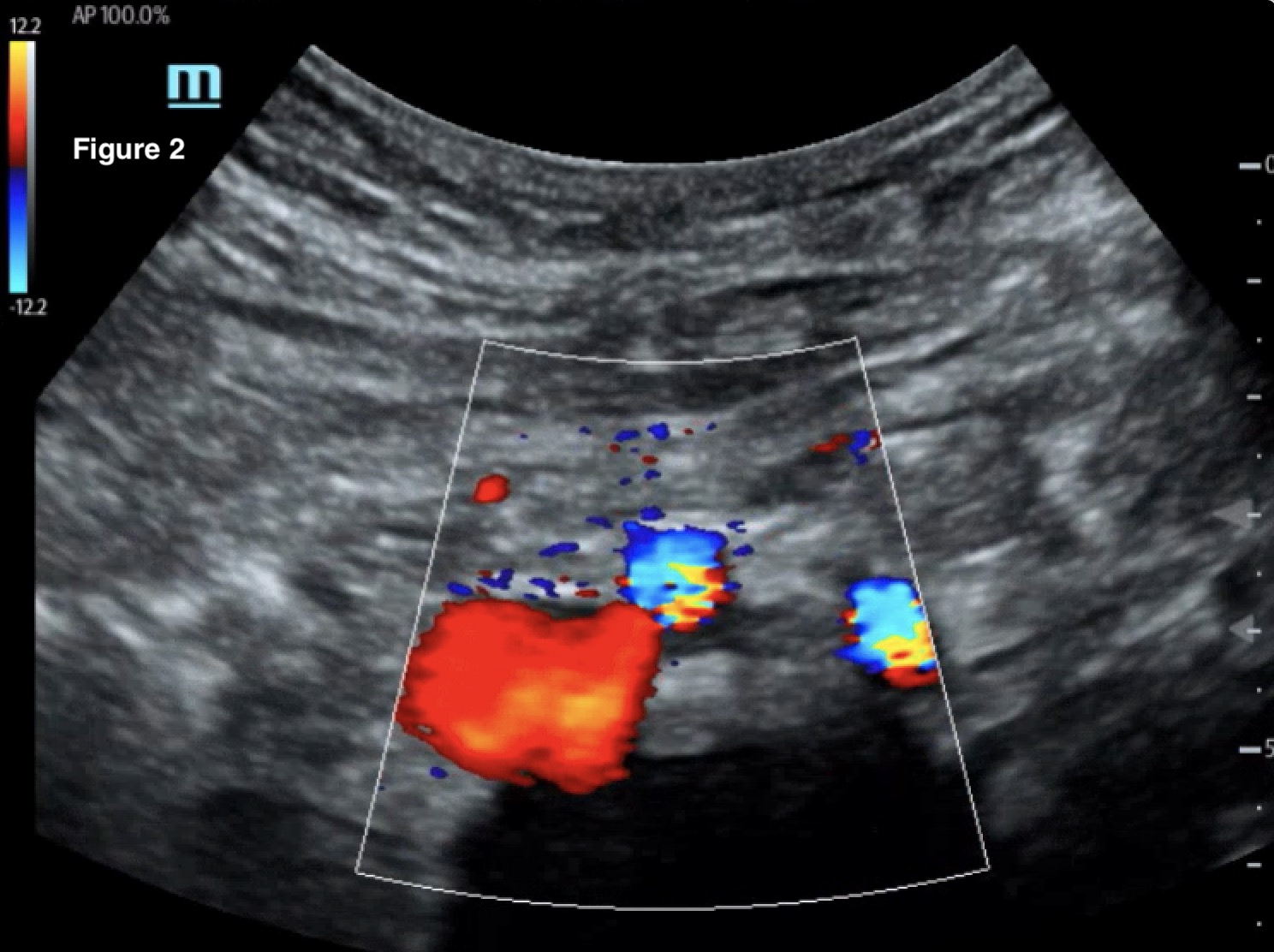

Supplement: Supplementary file 6 [file jetem-7-3-v14-supp6.jpeg]

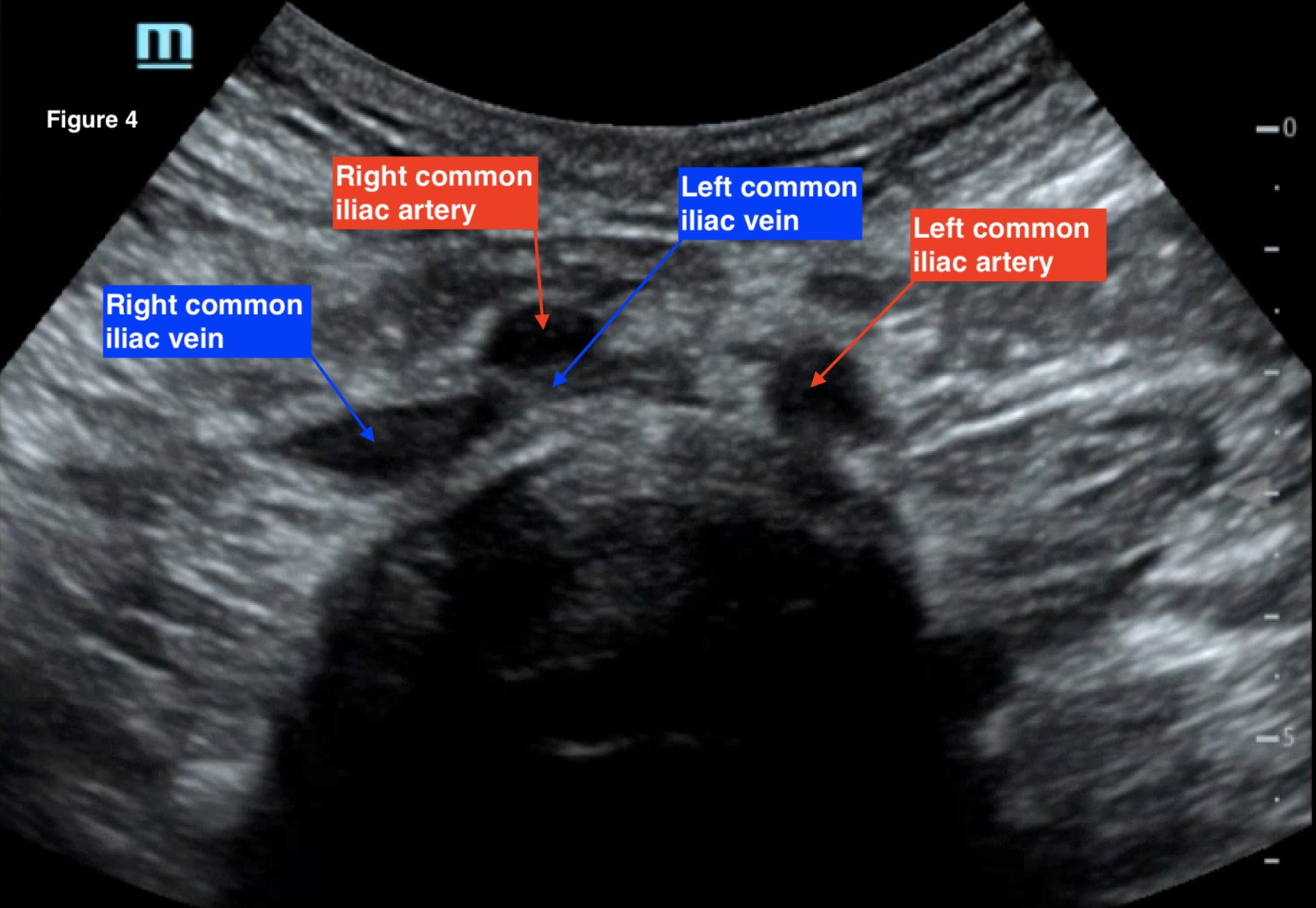

Supplement: Supplementary file 7 [file jetem-7-3-v14-supp7.jpeg]

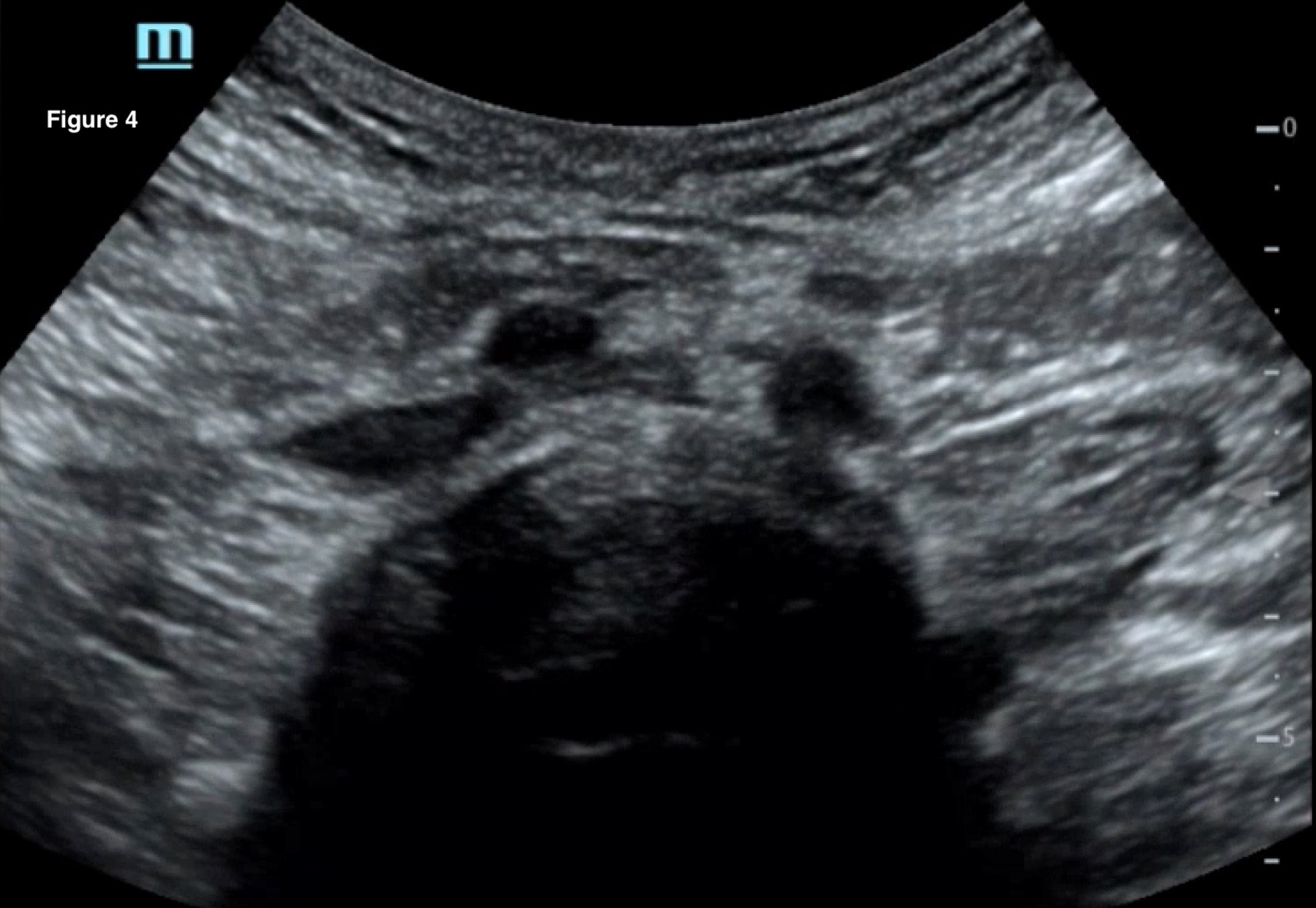

Supplement: Supplementary file 8 [file jetem-7-3-v14-supp8.jpeg]
